# Risk of Anaplastic Large Cell Lymphoma Following Postmastectomy Implant Reconstruction in Women With Breast Cancer and Ductal Carcinoma in Situ

**DOI:** 10.1001/jamanetworkopen.2022.43396

**Published:** 2022-11-22

**Authors:** Connor J. Kinslow, David M. DeStephano, Christine H. Rohde, Lisa A. Kachnic, Simon K. Cheng, Alfred I. Neugut, David P. Horowitz

**Affiliations:** 1 Department of Radiation Oncology, Vagelos College of Physicians and Surgeons, Columbia University, New York, New York; 2 Herbert Irving Comprehensive Cancer Center, Vagelos College of Physicians and Surgeons, Columbia University, New York, New York; 3 Division of Plastic and Reconstructive Surgery, Department of Surgery, Vagelos College of Physicians and Surgeons, Columbia University, New York, New York; 4 Department of Medicine, Vagelos College of Physicians and Surgeons, New York, New York; 5 Department of Epidemiology, Mailman School of Public Health, Columbia University, New York, New York

## Abstract

This cohort study examines the risk of anaplastic large cell lymphoma (ALCL) following postmastectomy implant reconstruction among US women with breast cancer and ductal carcinoma in situ (DCIS).

## Introduction

Approximately 291 000 and 51 000 cases of breast cancer and ductal carcinoma in situ, respectively, are diagnosed in the US annually, the majority of whom undergo local curative surgery, including mastectomy with implant-based reconstruction. National and regulatory guidelines recommend that patients eligible for reconstruction be counseled on the association of breast implants with subsequent anaplastic large cell lymphoma (ALCL) as part of routine informed consent.^1,2^ Several series of patients with breast ALCL have found overrepresentation of patients who received reconstructive vs cosmetic surgery, leading some investigators to suggest that ALCL may be more common in women with breast cancer or a genetic risk for breast cancer.^3^ However, there is a paucity of data available to guide clinicians in counseling patients on the relative and absolute risks of ALCL after implant-based reconstruction. We previously reported that the incidence of ALCL is increasing rapidly in the US.^4^ Here, we report on the risk of breast ALCL in a population of women who have undergone implant reconstruction.

## Methods

We identified women who underwent cancer-directed mastectomy with implant reconstruction for any tumor within the breast, diagnosed from 2000 to 2018, using the Surveillance, Epidemiology, and End Results (SEER) 17 database, excluding women with less than 12 months of follow-up. Participants were assessed for pathologically confirmed primary breast ALCL (*ICD-O-3* code 9714) through December 2019 until death, loss to follow-up, or end of study, with a latency exclusion period of 2 months from diagnosis. In a sensitivity analysis, we included cases of T-cell lymphoma, not otherwise specified (T-NOS; *ICD-O-3* code 9702).^4^ Multiple primary-standardized incidence ratios (SIRs) were used to compare the number of observed vs expected cases diagnosed in the study population based on US incidence rates derived from the US general female population in SEER 17, adjusted for age, race, and year of diagnosis. The prevalence of breast implants in US women is approximately 3.6% to 4.9%.^3,5^ Statistical analyses were conducted using SEER*Stat Version 8.3.9 (National Cancer Institute) and RStudio version 1.4.1106 (R Project for Statistical Computing) software. This study was exempt from review by the Columbia University institutional review board. This report follows the STROBE reporting guideline for observational studies. Two-sided *P* < .05 was considered statistically significant.

## Results

We identified 56 784 women who received postmastectomy implant reconstruction (median [IQR] age range, 50-54 [40-59] years; 18% with in situ disease and 72% with invasive disease). The sample included 7% Asian or Pacific Islander women, 8% Black women, and 84% White women. The median (IQR) follow-up time was 81 (46-125) months, including 15 765 women with follow-up times of at least 10 years. Five cases of breast ALCL were diagnosed over 421 223 person-years. The observed vs expected incidence rates were 11.9 vs 0.3 per million persons per year (excess risk 11.6 cases/million/y, SIR = 40.9 [95% CI, 13.3-95.5]) (Table). There was 1 additional T-NOS diagnosed, with a similar excess risk (13.8 cases/million/y) and SIR (34.8 [95% CI, 12.8-75.8]).

**Table.** Relative and Absolute Risk of Breast ALCL and ALCL/T-NOS after Implant Reconstruction

Histology	Incidence, cases/million/y^a^		Excess risk, cases/million/y^a^	SIR (95% CI)
	Observed	Expected		
ALCL	11.9	0.29	11.6	40.9 (13.3-95.5)
ALCL/T-NOS	14.2	0.41	13.8	34.8 (12.8-75.8)

Abbreviations: ALCL, anaplastic large cell lymphoma; T-NOS, T-cell lymphoma, not otherwise specified; SIR, standardized incidence ratio.

^a^ Incidence rates are derived from the SEER 17 database, 2000-2019, with reference rates calculated from the US Census Bureau, adjusted for age, sex, race, and year of diagnosis.

## Discussion

To our knowledge, we report the first population-based estimate of the risk of ALCL after implant-based reconstruction. Although the relative risk is significantly increased, the absolute risk remains extremely low. Furthermore, our risk estimates are within the range of reported literature for cosmetic or reconstructive implants. Breast implant–associated ALCL was not readily diagnosed in practice before 2008. The risk of ALCL will likely increase with longer follow-up, as the incidence rate is rapidly increasing in the US.^4^

Limitations of this study include retrospective analysis of registry data that depends on accurate reporting. We performed a sensitivity analysis, including cases of T-NOS, to account for underreporting. The latency time to develop secondary ALCL after implantation may be very long, and while our follow-up period was commensurate with other cohort studies, it likely underestimates the true incidence. Furthermore, we may underascertain cases due to migration from SEER registries. We did not account for implant removal or exchanges. Additionally, the confidence intervals of our risk estimates were wide.

Patients with breast cancer who are eligible for mastectomy should be counseled on the risks of breast ALCL after implant reconstruction. However, based on these results, we do not believe that women should be dissuaded from pursuing implant-based reconstruction due solely to the risk of ALCL. Specific brands of macrotextured implants with high-surface-area have been associated with higher rates of ALCL^6^ and may be avoided based on regulatory guidelines and clinical discretion.

## Notes

Supplement. Data Sharing Statement
