# Supplementary material for: Risk of Anaplastic Large Cell Lymphoma Following Postmastectomy Implant Reconstruction in Women With Breast Cancer and Ductal Carcinoma in Situ
Source: JAMA Netw Open. 2022 Nov 22;5(11):e2243396. doi: 10.1001/jamanetworkopen.2022.43396 (PMC9682428; doi:10.1001/jamanetworkopen.2022.43396)
Supplement: Supplement. — Data Sharing Statement [file jamanetwopen-e2243396-s001.pdf]

Data Sharing Statement

Kinslow CJ, DeStephano DM, Rohde CH, et al. Risk of anaplastic large cell lymphoma following postmastectomy implant reconstruction in women with breast cancer and ductal carcinoma in situ. *JAMA Netw Open*. 2022;5(11):e2243396. doi:10.1001/jamanetworkopen.2022.43396

Data
Data available: No

Additional Information
Explanation for why data not available: Data sharing per SEER policies. Data is available upon direct request to the NCI/SEER.
